# Characterising Wildlife Trade Market Supply-Demand Dynamics

**DOI:** 10.1371/journal.pone.0162972

**Published:** 2016-09-15

**Authors:** J. McNamara, M. Rowcliffe, G. Cowlishaw, J. S. Alexander, Y. Ntiamoa-Baidu, A. Brenya, E. J. Milner-Gulland

**Affiliations:** 1 Imperial College London, Division of Biology, Silwood Park Campus, Ascot, Berks, United Kingdom; 2 Institute of Zoology, Zoological Society of London, Regents Park, London, United Kingdom; 3 Centre for African Wetlands, University of Ghana, Legon, Accra, Ghana; 4 Ghana Wildlife Division, Forestry Commission, Accra, Ghana; 5 Department of Zoology, University of Oxford, Oxford, United Kingdom; U.S. Geological Survey, UNITED STATES

## Abstract

The trade in wildlife products can represent an important source of income for poor people, but also threaten wildlife locally, regionally and internationally. Bushmeat provides livelihoods for hunters, traders and sellers, protein to rural and urban consumers, and has depleted the populations of many tropical forest species. Management interventions can be targeted towards the consumers or suppliers of wildlife products. There has been a general assumption in the bushmeat literature that the urban trade is driven by consumer demand with hunters simply fulfilling this demand. Using the urban bushmeat trade in the city of Kumasi, Ghana, as a case study, we use a range of datasets to explore the processes driving the urban bushmeat trade. We characterise the nature of supply and demand by explicitly considering three market attributes: resource condition, hunter behaviour, and consumer behaviour. Our results suggest that bushmeat resources around Kumasi are becoming increasingly depleted and are unable to meet demand, that hunters move in and out of the trade independently of price signals generated by the market, and that, for the Kumasi bushmeat system, consumption levels are driven not by consumer choice but by shortfalls in supply and consequent price responses. Together, these results indicate that supply-side processes dominate the urban bushmeat trade in Kumasi. This suggests that future management interventions should focus on changing hunter behaviour, although complementary interventions targeting consumer demand are also likely to be necessary in the long term. Our approach represents a structured and repeatable method to assessing market dynamics in information-poor systems. The findings serve as a caution against assuming that wildlife markets are demand driven, and highlight the value of characterising market dynamics to inform appropriate management.

## Introduction

The trade in wildlife products, such as bushmeat, is one of the major drivers of extinctions worldwide [1]. As such, effective management of the wildlife trade is a pressing concern for conservation. In order to achieve this, conservationists need to understand the best points for management intervention; should interventions target the consumers, or suppliers, of wildlife products?

One approach that can inform this decision is to determine whether variation in price and quantity in wildlife markets are primarily driven by supply- or demand-side dynamics. For instance, in the case of bushmeat hunting, if demand-side dynamics are most important, interventions such as increasing the availability of alternative protein sources [2,3], changing consumer tastes [4], introducing farmed bushmeat [5,6], or trade regulation [7] may be most effective. Conversely, if supply-side dynamics predominate, then engaging with hunters to reduce their reliance on hunting through the development of alternative livelihoods [8,9], investment in human or social capital in hunting villages to reduce poverty and reliance on wildlife and improve social mobility [1,10], or stricter enforcement of hunting regulations [11] might be more appropriate.

Studies exploring the dynamics of wildlife markets have often assumed that the trade is dominated by demand side dynamics, “demand-driven”, i.e. that the relationship between price and quantity is based on maximising the utility of the consumer. Under this assumption, those supplying wildlife products, such as bushmeat hunters, will produce at a level which meets this demand [2,12,13]. This assumption is in agreement with traditional microeconomic theory [14,15]. However, in a challenge to this theory, Ghosh [16] hypothesized a case in which the supply curve was inelastic and unable to respond to a change in demand, due to resource limitation or some central control mechanism, such as rationing. Under this scenario, fluctuations in price and quantity are often more dependent on supply-side dynamics, or “supply driven”. Other situations where supply may be inelastic include markets with few barriers to entry, and where the opportunity costs of participation are influenced by the quality and availability of alternative income streams. Rising opportunity costs result in suppliers leaving the market to focus on other more lucrative income generators, while falling opportunity costs lead them to enter irrespective of market prices. Such supply-driven markets can be prone to considerable fluctuation in supply independently of changes in demand. The wildlife trade in general, and the bushmeat trade in particular, exhibits a number of characteristics associated with ‘supply-driven’ markets: limited exploitable resources, low barriers to entry (depending on the type of hunting: [17]), and variable opportunity costs because hunting is usually carried out as part of a diversified livelihood strategy [18].

Econometricians use market information to understand the processes that drive markets [19], but their approaches require detailed data that are rarely available in developing countries where the wildlife trade is frequently informal and monitoring capacity is low. To avoid the need for detailed, consistent, long-term market data on wildlife commodities and other variables likely to influence supply and demand (such as resource condition, production costs, consumer incomes, etc.), we develop a general framework, based on simple concepts from the economics literature, that describes the expected characteristics of a market under both supply- and demand-driven market regimes. Our framework is designed to accommodate the types of data often collected during small-scale or snapshot wildlife trade and livelihood surveys. It should therefore be applicable to a range of data-poor systems. It examines three attributes likely to be common to all wildlife and Non-Timber Forest Product (NTFP) markets: resource condition, supplier behaviour, and consumer behaviour (Table 1).

**Table 1:** Conceptual framework for evaluating the relative dominance of supply- or demand-side processes in wildlife trade markets. Note that the three attributes can operate independently, and no correlation between them is implied by the structure of the table.

Attribute	Demand-driven market predictions	Supply-driven market predictions
Supplier behaviour	Suppliers respond to price signals from the market, changing supply in response to price	Suppliers participate in the market independently of price signals generated by the market
Resource condition	Resource sufficient to meet demand (at least in short run)	Resources may be insufficient to meet demand
Consumer behaviour	Consumer choice defines patterns of consumption	Consumer choice constrained by resource availability and price

In this paper, we test the hypothesis that the bushmeat trade may be supply-driven. We focus on the urban trade in West Africa, using the city of Kumasi, Ghana, as a case study. To test this hypothesis, we use our framework to formulate a series of predictions for each market attribute. We then test these predictions using a combination of historical sources and primary field data (Table 2). On the basis of our findings, we draw conclusions about trade dynamics and therefore the potential effectiveness of different interventions for improving sustainability.

**Table 2:** Application of the research framework in Table 1 to test the hypothesis that the Kumasi bushmeat trade is primarily driven by processes related to supply.

Framework Attribute	Supply-Driven Market Predictions	Tests
Supplier (hunter) behaviour	1. Hunters move in and out of the market independently of price signals	Short-term (intra-annual) 1.1 Hunting activity seasonal, defined not by the price of bushmeat, but by other factors associated with hunters’ livelihoods, namely the agricultural seasons Long-term (inter-annual) 1.2 Participation in the trade varies independently of market signals (long-term average prices), and the value of bushmeat relative to the national minimum wage and the price of alternatives
Resource condition	2. Resources may be insufficient to meet demand	2.1 Average duration of hunt increasing 2.2 Catch per unit effort declining 2.3 Hunters report change in offtake towards less vulnerable groups such as rodents 2.4 Market data suggests an increase in the proportion of the trade represented by less vulnerable taxonomic groups
Consumer behaviour	3. Consumer choice constrained by resource availability and price	3.1 Frequency of bushmeat consumption in decline due to high prices 3.2 Evidence that consumers are switching to cheaper alternatives

## Methods

### Study site

Kumasi is Ghana’s second largest city, situated in the country’s Ashanti region. The city provides a valuable case study for examining the drivers of the bushmeat trade due to a long history of bushmeat research [17,18,20–22] which allows an historical perspective to be taken over three decades. The city is home to the Atwemonom bushmeat market, one of the oldest and largest bushmeat markets in Ghana, fed by the historically rich forests of the region [21]. Atwemonom (literally "place for fresh duiker meat") is the only formal market for fresh bushmeat in central Kumasi, thus the trade passing through this market may be considered indicative of the general trade in fresh bushmeat in the city. Spatial analysis of the Atwemonom catchment area confirmed that meat is sourced locally to the market [23]. Hunters generally transport meat to market personally rather than through middlemen [21,24] and consequently when considering supply-side dynamics we have chosen to focus solely on hunter behaviour and not considered the role of traders or middlemen. Hunting is regulated by the Wildlife Conservation Regulations [11,25], which impose a strict ban on hunting of all species except the grasscutter (*Thryonomys swinderianus*) between 1 August and 1 December each year, a period referred to as the “closed season”. For the remainder of the year, from December through to July, hunting is permitted for all species except those listed as schedule 1 in the Wildlife Conservation Regulations. The city is located in the Upper Guinea Forest Ecosystem, a biodiversity hotspot that has experienced severe degradation [26]. The overexploitation of wildlife populations in this region is widespread, but the bushmeat trade remains an important source of income and protein for many rural and urban households. Therefore the sustainable management of the trade is of high concern to both conservationists and development professionals.

### Ethics statement

The research was carried out in partnership and with the approval of the Ghana Wildlife Division and in accordance with the ethics guidelines of the Association of Social Anthropologists of the UK and Commonwealth. A meeting was held with community leaders prior to data collection to explain the purpose of the study and to request permission for the research. Prior to each individual questionnaire being conducted, the purpose of the research and content of the questionnaire were explained. It was explained that responses might be anonymously published in scientific literature. All participants provided informed consent to be interviewed and for their responses to be used in this way. Due to low levels of literacy, this consent was given verbally in the presence of both a member of the Ghana Wildlife Division and a member of the local community acting as a local guide and translator. All participants were over the age of 18. All data collected were anonymous to remove the chance of any participant being identified.

### Data Collection

**Fig 1:**
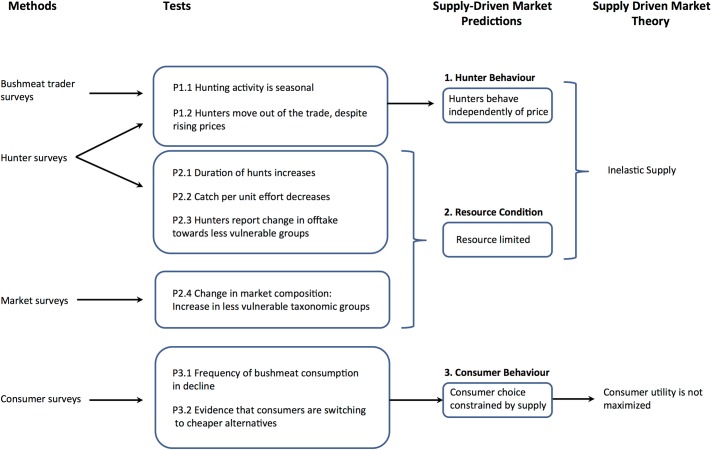
A conceptual diagram showing how the methods adopted in this study relate to the research framework (Tables 1 and 2) and underlying economic principles.

A combination of primary and secondary data is used in this study. Primary survey data were collected in May 2011 in both urban (Kumasi) and rural (villages around Kumasi) settings to gather information on hunter behaviour, resource condition, and consumer behaviour. These data were complemented by secondary data, taken from the literature on the bushmeat trade in and around Kumasi, to provide an overview of how these attributes have changed over time. A conceptual diagram that describes how the methods detailed below link back to the research framework presented in Tables 1 and 2 is presented in Fig 1.

### Primary Data

#### Rural surveys

Two rural communities close to Kumasi were surveyed: Jachie (6.568°, -1.521°), 20km to the southeast; and Kwaman (6.977°, -1.272°), 65km to the northeast. Both communities were known to be regular suppliers of bushmeat to the Kumasi market [24]. Prior to commencing formal surveys, focus groups were held with 8–10 individuals in each village. These groups focused on aspects of village life and livelihoods, including the agricultural calendar, and comprised senior members of local hunting associations, who were also heads of households and therefore well placed to answer questions about household behaviour. Information from the focus groups guided the more detailed one-to-one surveys conducted with hunters.

Semi-structured interviews (S1 Appendix) were held with hunters (Jachie N = 23, Kwaman N = 28) who were identified using three methods: during systematic household surveys [18]; through membership in local hunting associations; and through snowball sampling. Hunters were defined as those who identified hunting as a livelihood activity, whether for food or income. A detailed description of survey methods is published in Alexander et al. [18].

#### Urban surveys

Urban surveys were conducted in four market areas in Kumasi’s central business district: Atwemonom (a specialist market selling only fresh bushmeat), Central Market, Racecourse Market, and Kejetia (three general markets selling a variety of goods and services, but not fresh bushmeat). Surveys were of three types. First, consumer surveys were conducted as semi-structured interviews with the general public based on a random sampling technique where members of the public who were happy to be interviewed were questioned. These recorded protein preferences and the regularity with which different proteins were consumed (bushmeat, farmed meat, fish). These surveys were conducted in Central Market (N = 35), Racecourse Market (N = 30), Kejetia (N = 16), and the streets in between (N = 20), and were short and simple to encourage participation (6–12 questions depending on whether an individual consumed bushmeat; S2 Appendix). Second, bushmeat trader surveys were conducted as semi-structured interviews with wholesale traders in Atwemonom and the owners of chopbars (small street restaurants selling bushmeat dishes) across the city (S3 Appendix). These surveys recorded information on bushmeat prices, availability and seasonal trends (wholesaler N = 11, chopbar N = 6). Third, a market survey was conducted in Atwemonom over one week in May 2011. The timing of this survey was chosen to facilitate comparison with a previous survey [21]. Data were gathered on price, weight and species traded. In addition, fish and livestock prices were collected in the Central and Racecourse Markets. Raw data for all surveys conducted as part of this study are available in S4 Appendix.

### Secondary data

Historical data on the bushmeat trade in this region are available for six different years over a 30-year period, in 1982 [20], 1990 [21], 1993 [27], 1995 [17], 1997 [28] and 2002 [29]. Additional data on economic indices, namely exchange rates, the consumer price index and national minimum wage were collected from international and national institutions including the International Monetary Fund, World Bank and Ghana Statistical Service.

### Data analysis and tests of predictions

#### Hunter behavior

Our first prediction, that hunters move in and out of the market independently of price signals (Table 2), was assessed through both short-term and long-term patterns in hunting behaviour. The seasonality of hunting had been confirmed in previous research visits to the study sites and the literature [17,24]. To examine short-term trends (prediction 1.1), we asked hunters how they allocated their time throughout the year. Variation in hunting pressure was quantified by asking hunters to name the months when they hunted most (the peak months) and the months when they hunted least (the low months). To test whether the effort exerted was different between seasons, hunters were asked to state their weekly expectations for how many trips they might engage in and how many animals they might catch in both low and high seasons. These questions were also asked in 2002 [29], allowing a comparison over time. Wilcoxon tests were used to analyse seasonal differences. The hunters’ motivations for acting as they did were explored through the hunting surveys. Seasonal trends reported by hunters were cross-referenced with information from the bushmeat trader surveys.

Long-term participation in hunting (prediction 1.2) was explored by contrasting the proportion of active hunting households in a community in three different years: 1995, 2002 and 2011. These data were compared to changes in the real price of bushmeat over the same period. An estimate of the relative value of bushmeat was constructed by comparing the real price of bushmeat (deflated with consumer prices index using 2004 as a baseline) to the national minimum wage, the real price of cocoa (the most important cash crop in the region, and thus a rough proxy for agricultural income) and the real price of substitute goods (in this case, fish, the most commonly-consumed protein). This “value estimation” is intended to place changes in bushmeat prices in the context of changes in other key price and cost indices to determine how the relative value associated with the trade has changed.

#### Resource condition

Our second prediction, that resources show signs of depletion (Table 2), was assessed through four tests involving two metrics, namely catch per unit effort and trade composition.

Estimates of the length of the average hunting trip (prediction 2.1) were available from a number of studies in the Ashanti region in four different years: 1982, 1993, 2002 and 2011. Catch data were only available from one comparable study in 2002. Thus catch per unit effort (prediction 2.2), described in terms of the average number of animals caught per hour spent hunting, is presented for 2002 and 2011. The change in the proportion of hunters in Kwaman who believed that there had been a decline in bushmeat offtake is presented between 1995 [17] and 2011.

Changes in the composition of the trade (prediction 2.4) were first examined using data collected in the Atwemonom market in 1990 [21] and 2011 (primary data). This information was subsequently cross-referenced with firsthand hunter reports, gathered during hunter surveys collected in 2011, to validate the degree to which hunters corroborated the trends in the market data (prediction 2.3). As part of this process, hunters were asked to list those species that they caught frequently (categorised as "present"), and those that they used to catch frequently but now caught very infrequently or not at all (categorised as "absent"). This information was used to calculate the proportion of hunters reporting that a particular species was “present” or “absent”. Thus an estimation of the relative scarcity of each species, from the perspective of the hunter, was made. Hunters’ responses were unrestricted and they were free to list as many species as they wished (although in reality this number never exceeded four per category).

#### Consumer behavior

Our third prediction, that consumer choice is constrained by resource availability and price (Table 2), was assessed through five tests involving data from three different years: 1990, 1997 and 2011. Frequency of bushmeat consumption (prediction 3.1) was measured using three metrics: (1) the proportion of consumers who eat any bushmeat, (2) the frequency of bushmeat consumption and (3) the proportion of consumers who used to eat bushmeat but no longer do so. Evidence that consumers are switching to cheaper alternatives (prediction 3.2) was assessed using two metrics: (1) changes in preference for bushmeat and fish and (2) consumers’ willingness to pay more for the bushmeat they consume. Further, in 2011 we asked consumers who reported no longer eating bushmeat (but who had done so in the past) why they made this choice, to ascertain whether price, preference, health, religion or some other factor played a role.

## Results

### Hunter behaviour

#### Short-term, intra-annual

**Fig 2:**
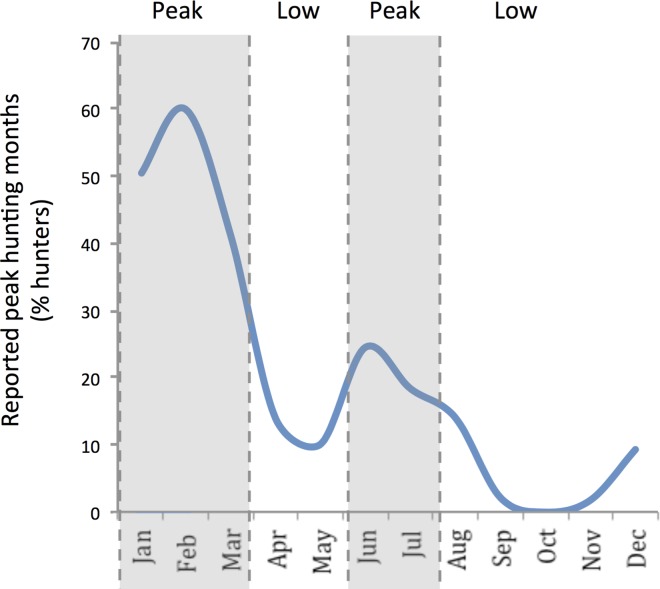
Percentage of hunters in Jachie and Kwaman villages who associate any given month as being part of their peak hunting season. Inferred peak and low seasons indicated by shading. N = 51.

Hunting was a strongly seasonal activity; only 22% of those surveyed in 2011 reported hunting all year round. All hunters reported a peak and low season for hunting during the year (Fig 2). The majority of hunters, 80%, hunt primarily for income and view it as a commercial enterprise. Hunter effort and success, measured in trips per week and animals caught per week respectively, were both higher in the peak season (Kwaman, trips: V = 196.5, P = 0.0006; animals: V = 325, P = 1.29x10^-5^; Jachie, trips: V = 140, P = 0.003; animals: V = 120, P = 0.0007). Two previous studies supported this seasonal trend, reporting that Atwemonom market supplies peaked between January and March, and declined in April and May before rising again in June [17,21]. A study in 1990 [21] found prices to be higher in the low season: the relative price mark-ups for the four main species were 67% (grasscutter), 29% (Maxwell's duiker *Philantomba maxwellii*), 60% (black duiker, *Cephalophus niger*) and 114% (bushbuck *Tragelaphus scriptus*).

In the 2011 survey, all hunters gained income from other sources as well as hunting. Almost all hunters (94%) engaged in agriculture to some degree, and the vast majority (80%) stated that agricultural commitments were the primary reason they chose to hunt more in the peak hunting season. Of these, 47% chose the peak season because of fewer labour commitments associated with harvesting and planting at this time, while 33% chose it because of the need to protect their crops, in particular maize, from pests. Both of these observations highlight the primacy of agriculture in livelihood decision-making. The remaining 20% linked their choice of the peak season to favourable environmental conditions, as it aligns with the dry season when undergrowth in the forest is minimal and animals are easier to see. No hunter mentioned the price of bushmeat or market incentives as a reason for allocating effort as they did. The interviews with bushmeat traders confirmed seasonal trade fluctuations in line with the hunters’ allocation of effort.

#### Long-term, inter-annual

Engagement in hunting appears to have declined over the last decade. Household surveys in communities around Kumasi in 1990 indicated that 14% of households were involved with hunting [21]. This aligns well with the surveys conducted in 2002, indicating that approximately 15% of households were involved in hunting [29]. However, surveys in 2011 found that only 4% of households were engaged in hunting [18].

Comparisons of proportions might hide an absolute increase or decrease in hunting households, if the number of households in the community has changed. Household censuses for the surveyed communities were unavailable, so it is not possible to represent the change in household participation in absolute numbers. However, as a coarse comparison, between 1999 and 2012 the rural population in Ghana increased by 6%, less than the 11% proportional decline in hunting households in Jachie and Kwaman between 2002 and 2011 [30,31].

In contrast, over a comparable period, bushmeat has not only been the most expensive protein available in markets in the region [6,21,22], but its value relative to other proteins has increased. In 1990 fresh grasscutter meat was on average 39% more expensive than beef and 51% more expensive than goat [21]. In 2011, a kilo of grasscutter meat was 108% more than a kilo of beef, 67% more than a kilo of goat and 488% more than a kilo of fresh sardines. Similarly, analysis of the marginal increase in bushmeat prices between 1990 and 2011 (using the wholesale price of a single grasscutter carcass as an indicator) shows that the real price of bushmeat increased by 313%. By contrast, over the same period, the national minimum wage increased by 61% [32]; the real price of cocoa, the main cash crop in the area and thus an indicator of agricultural income, rose by 153% [33]; and the real price of herring, the most commonly consumed protein according to our survey of Kumasi consumers, by 180%.

In 2011, the price a hunter could expect for a single grasscutter carcass was 57% more than a worker on the national minimum wage could expect to earn in a week. In 1990, it was 30% less. According to the Ghana Statistical Service, inflation between 1990 and 2011 was 4,930%. Over the same period, the raw price of the average grasscutter carcass increased by four times this rate. While this comparison presents only prices, ignoring external production costs such as fuel that might impact profit margins, it highlights how the consistent rise in bushmeat price has exceeded price rises of similar commodities. The implication is that the relative value of bushmeat is greater in 2011 than it was in 1990, but that participation in the trade has declined.

These findings suggest that at both short-run and long-run timescales hunters appear to behave independently of price (in support of predictions 1.1 and 1.2). Seasonal participation is reported to be driven largely by external livelihood commitments, particularly agriculture, with hunters reducing hunting effort during periods where meat prices are higher, while long-term engagement appears to be in decline. Both behaviours appear contrary to price signals generated by the market.

### Resource condition

#### Trade composition

**Fig 3:**
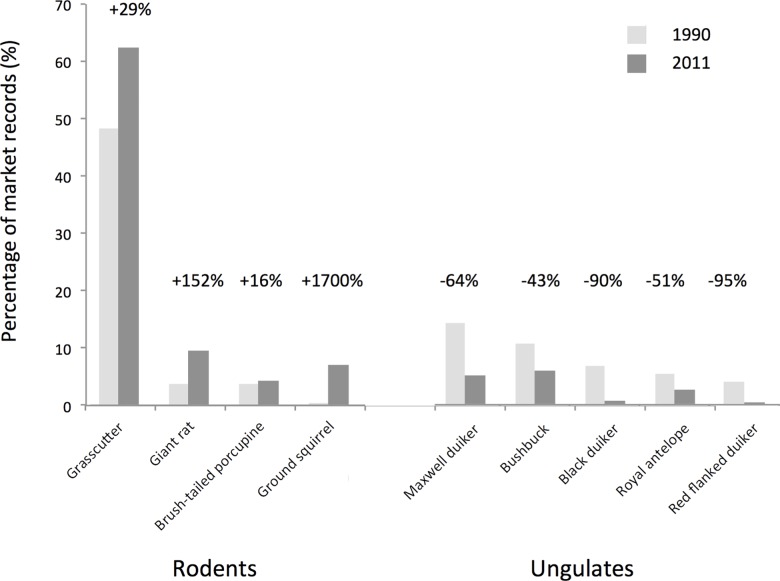
Comparison of percentage market share (measured in terms of total carcasses) in 1990 (total carcasses = 771) and 2011 (total carcasses = 417) for the top nine most traded species in the market (representing >95% of the market in both years). Numbers above the bars represent the change in market share between years. The 1990 data [21] are derived from surveys conducted over 12 days in April, 9 days in May and 6 in June. The 2011 data (primary data) are derived from surveys conducted over 6 days in May. Species are ordered by their relative contributions in 1990 within their taxonomic group (rodents or ungulates).

The historical data suggest that the profile of the trade entering Atwemonom market has been relatively stable over the past 20 years in terms of species type, with the bulk being represented by nine species of ungulate and rodent. Closer inspection, however, suggests an underlying shift in composition. All four rodent species increased their market share, while all five ungulate species declined (Fig 3). This change was reflected in an increase in the ratio of rodents to ungulates, from 1.4 in 1990 to 5.8 in 2011. Additionally, larger species (>5kg) were significantly more likely to lose market share than smaller species (Fishers exact test for a 2x2 contingency table: p = 0.04). A comparison of 1990 and 2011 market surveys, and all data collected in 2011, are presented in S5 and S6 Appendices respectively.

**Fig 4:**
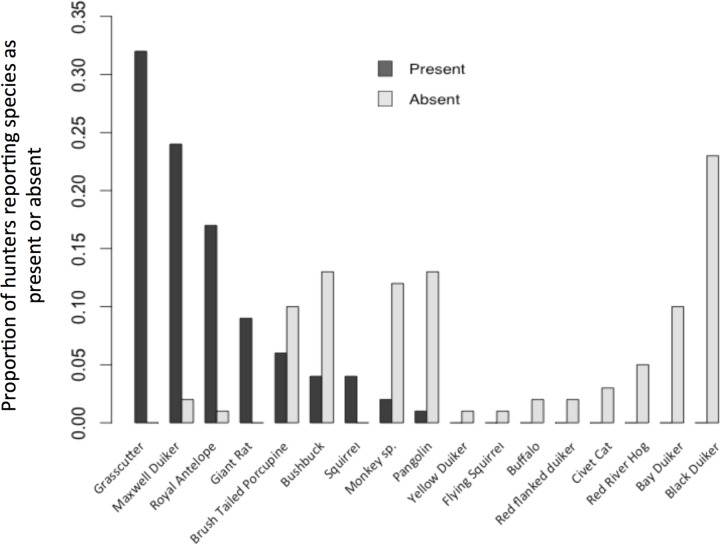
Proportion of hunter reports citing particular species as being present or absent in their catch. ‘Present’ refers to species caught frequently; ‘Absent’ refers to species that used to be caught frequently but are now rare or absent entirely. Species are ordered by decreasing proportion of ‘Present’ reports, followed by increasing proportion of ‘Absent’ reports.

Hunter observations corroborate these trends. The seven most common species in our 2011 market survey were all identified as being regularly caught by hunters. The most common species, the grasscutter (2011 market share: 63%), was the species most frequently cited as being caught by hunters. Furthermore the black duiker, which has suffered a notable decline in market share (90% decline), was the species most commonly reported by hunters as previously present but now absent (Fig 4). The other species to show a notable decline, the red-flanked duiker *Cephalophus rufilatus* (95% decline), was discussed only in terms of its absence. A breakdown of hunter catch reports ordered by body mass is presented in S7 Appendix.

#### Catch per unit effort

There was unanimous consensus among hunters that bushmeat species were in decline and that it was necessary to hunt for longer and travel further than in the past (S8 Appendix). The average time spent hunting by hunters in the Kumasi area appears to have increased by almost 114% in the last 30 years, from 3.6 hours per hunt in 1982 to 7.7 hours in 2011 [18]. There was also a decline in catch per unit effort of 46%, from 0.35 animals per hour (SD = 0.15) in 2002 [29] to 0.19 (SD = 0.12) in 2011 (t = -test: t = 0.73, d.f. = 73, p = 0.02). Further evidence that resources may have been declining for some years comes from a 1995 study, that reported that 98% of hunters perceived bushmeat hunting success to be in decline, and 70% believed this to be due to dwindling wildlife populations [17]. A similar study in 2011 found that all hunters reported a decline in success and increasing difficulty in securing a successful catch [18].

These findings suggest that the overall availability of bushmeat and its species composition are in line with expectations under long-term depletion, from the perspectives both of the market and hunter. This supports predictions 2.3 and 2.4. An increase in the length of each hunt, and declines in catch per unit effort (in support of predictions 2.1 and 2.2), suggest this shift may be associated with reduced supply, questioning the degree to which supply is adequate to meet demand.

### Consumer behaviour

Comparison of historical consumer survey data from Kumasi and the surrounding communities suggests that between 1990 and 2011 there has been a significant decline in the proportion of people who eat bushmeat (Z = 3.04, P < 0.005). There has also been a significant decline in the proportion of consumers who report eating bushmeat daily (Z = 7.4, P < 0.005), and a significant increase in the number of consumers who used to eat bushmeat but no longer do (Z = -1.95, P = 0.02) (Table 3). In 2011, all 18 respondents who used to eat bushmeat in the past cited cost as the reason. This despite the fact that 80% of consumers surveyed in 2011 stated that they felt consuming bushmeat was culturally important.

**Table 3:** Comparison of patterns of bushmeat consumption between 1990 and 2011.

**Consumption characteristic (%)**	1990* **(N = 101)**	2011** **(N = 101)**
Eat bushmeat (any)	86	69
Consume bushmeat (daily)	52	6
No longer consume bushmeat	9	18
Willing to pay more for bushmeat	55	32
**Stated preferences (%)**	1997*** **(N = 144)**	2011** **(N = 100)**
Bushmeat	26	14
Fish	30	61

* Study area: Kumasi [21]

** Primary data collected during consumer surveys, May–June 2011, study area: Kumasi and surrounding communities.

*** Study area: Kumasi [28].

Evidence that the high price of bushmeat discourages consumption has been reported previously in Ghana. A 1993 study of three communities in southern Ghana found that scarcity and price were the most frequently quoted reasons for not eating more bushmeat [27]. By contrast consumer preferences appear to be increasingly favoring less expensive proteins. Between 1997 and 2011, there has been a significant decrease in stated preference for bushmeat, (Z = 2.27, P = 0.01) accompanied by a significant increase in preference for fish (Z = -4.83, P < 0.005). This observation is accompanied by a significant decline in consumers’ willingness to pay more for the bushmeat they consume (Z = 3.43, P = <0.005) suggesting that observed increases in the real price of bushmeat are influencing consumer choice.

These findings suggest that the frequency of bushmeat consumption by individuals is in decline due to high prices (in support of prediction 3.1). Associated with this pattern has been a reduction in preference for, and in willingness to pay more for, bushmeat. This is accompanied by a growing preference for fish, suggesting consumers are switching to cheaper alternatives (in support of prediction 3.2). These phenomena suggest that the high price of bushmeat is increasingly pricing individual consumers out of the market, suggesting that supply is failing to keep track with demand. This is in line with our prediction that consumer choice is constrained by a supply-limited market regime.

## Discussion

The tests of our predictions provide good evidence in support of our hypothesis that the trade around Kumasi is dominated by supply-side characteristics, as characterised in our framework (Table 1; Fig 1). This framework relies on two simple concepts from economic theory. Firstly, that in a supply-side dominated market, supply is inelastic; and secondly, that as a consequence of a short-fall in supply, consumer choice is constrained by resource availability and price, and therefore consumer utility is not maximised.

### Supply Inelasticity

#### Hunter behaviour–opportunity costs

The seasonal correlation between hunting effort and agriculture (prediction 1.1) is in agreement with the findings of other studies in the region [34]. This highlights the importance of taking into account hunters’ broader livelihood portfolios when considering their sensitivity to opportunity costs. Studies in central Africa have shown that hunters are sensitive to changing economic incentives, increasing effort in periods of recession and high unemployment [35] and choosing better paid alternative employment when opportunities present themselves [36]. It would be interesting to examine how different livelihood strategies affect hunters’ sensitivity to opportunity costs. For example, if they were involved in business that provided year-round income support, such as running a bar, investment of time in hunting might be less seasonally forced than is the case for agriculture.

#### Hunter behaviour–barriers to entry

Another determinant of an individual's sensitivity to opportunity costs is the barriers to entry to the focal livelihood. If these are high, individuals may be slower to respond to changing economic incentives. The primary expense for a hunter wishing to enter the bushmeat trade is the purchase of a firearm. Although often produced locally by village blacksmiths, the expense of a firearm is not negligible. A 1995 study in the region concluded that although firearms are expensive and likely to act as a barrier to those wishing to enter the trade, once purchased the ongoing costs of participation (cartridges and batteries) are relatively low, particularly for commercial hunters where the price of bushmeat far outweighs the marginal equipment costs associated with these items [17]. Thus, for those already engaged in the trade, it is likely to be fairly easy to adapt effort efficiently in response to changing opportunity costs. For new entrants, a firearm may represent a significant barrier to entry.

Long-term decline in hunting participation (prediction 1.2) could suggest that despite the increasing value of bushmeat, the resource base is in such poor condition that it is simply not profitable to harvest bushmeat intensively (as indicated by the significant decline in CPUE). A potential driver of declining CPUE, not quantified in this study, is production costs. Due to the artisanal nature of firearm production, data were not available on firearms costs. Firearm ownership is not a pre-requisite for hunting however. Cheaper options such as snares and dogs are alternatives [18]. In addition to firearms, another significant cost of participating in the commercial trade is transport to market [37,38]. Hunters generally use local transport to bring meat to market. Transport costs vary according to the distance, species of animal being transported and personal preference of the driver. Consequently, transport costs were not quantified, but an important consideration in this regard is the strong client-patron relationship in operation in the Atwemonom market, where traders assist hunters with short-term loans for equipment and transport, which can be repaid in meat. Furthermore, hunters surveyed around Kumasi indicate that they are able to incorporate the costs of transport into the wholesale trade price at market. Thus there are existing local finance schemes to help hunters with capital expenditure needed to hunt, and while transport is likely to be a significant determinant of price, it is unlikely to influence decisions on whether or not to participate in the trade.

Another explanation for a fall in hunting participation is an increase in school attendance among young men. Between 1999 and 2012 there was a marked increase in the proportion of young men in the rural forest zone attending school, from 41% to 95% of those aged between 16 and 18 and from 11% to 93% at ages 19–25 [30,31]. Higher standards of education may enable youths to seek higher paying employment, either locally or within cities, and this has been associated with an increased willingness to leave the bushmeat trade in times of difficulty [24].

#### Resource condition

We have interpreted the observed changes in trade composition both in markets and among hunters (predictions 2.4 and 2.3 respectively) as signs of resource depletion. However, our data on hunter effort and catch success rates rely on recall, and thus may be biased. We have attempted to limit this bias by taking evidence from different sources and time periods and, where possible, cross-referencing the findings with complementary sources such as trader reports. Another potentially significant driver of the change in composition of species traded on the market is land-use change. Between 1986 and 2002 the Kumasi bushmeat catchment area saw a reduction in areas of closed canopy forest outside reserves of 47%. The region is increasingly influenced by human disturbance, creating habitats where generalist species such as rodents, in particular the grasscutter, and certain antelope species such as Maxwell’s duiker (*Philantomba maxwellii) [39],* are better able to persist than forest-dependent species including antelope species such as the Black duiker (*Cephalophus niger*) and arboreal primates. This shift in land-use correlates with an increase in rodents traded on the urban bushmeat market [23]. Finally, previous studies have demonstrated that middlemen and traders can have significant influence on determining what meat makes it to market [40]. The fact that most hunters transport their own meat to market probably reduces the potential influence of similar processes in this system. However, such processes cannot be ruled out, and further research to consider how the supply chain operates could add valuable information for understanding market dynamics.

### Consumer behaviour

Contrary to demand-driven regimes, where the relationship between price and quantity is based on a set of choices that maximises the utility of the consumer [14], markets dominated by supply-side dynamics may be characterised by shortfalls in supply, inflated prices or both. It was not possible with the datasets available for this study to formally estimate demand elasticity for bushmeat or cross-price elasticities for alternative proteins, however shifts in consumption patterns reported in the consumer survey are in line with the findings of other studies that have explored these dynamics. Two previous studies, in the Serengeti region, have demonstrated that bushmeat demand is sensitive both to its own price (elasticity of demand) and the price of alternatives (cross-price elasticity of demand) [3,41]. Our findings provide qualitative evidence of a similar process. With the marginal real price of bushmeat increasing at a rate above inflation, and at a greater rate than alternative proteins, consumers in Kumasi appear to be reducing their frequency of consumption of bushmeat while favouring cheaper alternatives.

Our results do not allow us to draw conclusions about how the overall quantity of bushmeat demanded has changed, nor about any changes that may have taken place in the shape of the demand curve. Rather, they provide evidence that individual frequency of consumption is in decline due to high bushmeat prices as consumers are unable to afford bushmeat. This implies that the system has effectively moved along the demand curve, shifting to less meat being demanded because it is on sale at a higher price. This suggests that there is unmet latent demand in the system.

Further, data gathered from hunters and the commercial market provide persuasive evidence of depletion. If true, this implies that changing patterns of bushmeat consumption are likely to be heavily influenced by shortfalls in supply and the associated price increase. However, there is also evidence that the absolute number of bushmeat consumers in the city has increased. Population census data for Kumasi shows that between 2000 and 2010 the population of the city increased by 25%, while between 1984 and 2010 it increased by 114% [30,31]. This growth is likely to be adding upward pressure to demand, may offset the observed reduction in individual levels of consumption, and may in part explain the significant price rises observed on the market in recent years [23].

This has two implications. Firstly, that bushmeat species are highly depleted in the Kumasi region, particularly forest-dwelling species; this suggests conservation action is needed if these species are to persist in the region. Secondly, a cautionary note, that unfulfilled demand for bushmeat is likely to remain in the market. Although our data do not allow us to estimate this latent demand, the fact that such demand probably exists suggests that were the populations of bushmeat species to recover and catches improve, hunters would probably have little difficulty in selling their catch, albeit at a slightly lower price. On the other hand, previous research has highlighted that actual consumption and preferences are linked [42,43] and it may be that bushmeat, both as a livelihood and a food source, is already phasing out in this region, as a result of supply-side dynamics. Without more detailed information on how demand for bushmeat has changed, it is hard to draw definitive conclusions about the underlying drivers of the decline in bushmeat consumption observed in our study. Further research on consumer demand should be a priority to better to understand these processes and make predictions about future sustainability.

### Management implications

Hunting regulations that protect vulnerable species and set quotas on offtake are already in place through the Wildlife Conservation Regulations [11]. However, they are weakly enforced and, judging by historic declines in wildlife in the region, have a poor track record of success [44–49]. An alternative is to promote the benefits of the farm-bush matrix (from which many valuable species such as the grasscutter are harvested) as a source of livelihood resilience, for example by supplying evidence to governments of the additional economic productivity that can be achieved from wildlife harvests from low intensity agricultural systems [23]. Combined with local initiatives to promote wildlife-friendly farming practices, this approach could lead to better co-existence of resilient bushmeat species, such as grasscutters, with agricultural production. This focus on promoting sustainable and diversified livelihoods in rural areas [9] does not preclude improvement of institutions managing the region's remaining areas of protected forest, or enforcement of laws prohibiting the trade in vulnerable species.

Studies have shown that fishers from poorer households are less likely to exit a declining fishery [50]. Similarly, hunters’ willingness to stop hunting in communities around Kumasi has been linked to educational level as well as household wealth [24]. Understanding the socioeconomic profile of resource users, to better understand the underlying drivers of hunting and its cessation, should be a priority.

While this study highlights the importance of supply-side interventions, it should not be interpreted as suggesting demand-side interventions can be ignored. Both supply and demand interact to set price and quantity, and complementary measures need to be taken to ensure latent urban demand is reduced, thus limiting the incentive for new actors to enter to the trade. For example, measures could be taken to support a switch in consumer preferences to less vulnerable species, such as through investment in farm-reared stock to provide a sustainable supply [51–53].

Our study acts as a caution that wildlife trade markets should not be assumed to be demand-driven without evidence, however convenient this assumption may be for bio-economic modelling. Markets characterised by resource scarcity, where ecological constraints place a hard limit on harvests, may be the most likely to exhibit supply-driven characteristics, but resource users' livelihood portfolios (relating to opportunity costs) and the barriers to entry, are also important determinants of market dynamics. Further, if wildlife resources were to recover, or the wider rural economy of the region were to collapse, commercial hunters may be inclined to revert to full-time hunting activities to meet latent demand among those consumers who had previously been priced out of the market, resulting in the system reverting from a supply- to demand-dominated regime. The conservation implications could be severe, and require responses such as law enforcement at the consumer and supplier ends, together with action to support farming livelihoods.

The framework and methodology presented in this work provides a structured approach to developing and testing predictions which can indicate the degree to which supply or demand drivers predominate in a given system, based on an integrated analysis of information of a type which is often readily available. Making use of historical data, triangulating information from a range of actors in a commodity chain, and ensuring that new surveys are comparable with previous datasets where possible, can enable researchers to characterise market dynamics, albeit roughly, even when long time-series are not available. This can be enough to inform management decision-making in the absence of capacity for sophisticated monitoring [54]. We hope that this approach will be useful to those aiming to intervene to make the trade in wildlife and other non-timber forest products more sustainable.

## Supporting Information

S1 Appendix Hunter survey instruments. (DOCX)

S2 Appendix Urban consumer survey instrument. (DOCX)

S3 Appendix Trader survey instrument. (DOCX)

S4 Appendix Raw survey data. (XLSX)

S5 Appendix A comparison of 1990 and 2011 bushmeat market surveys. (DOCX)

S6 Appendix Summary of the one-week survey of the Atwemonom bushmeat market in June 2011. (DOCX)

S7 Appendix Proportion of hunter reports citing particular species as being present or absent in their catch. (PDF)

S8 Appendix Summary of average hunting trip length and catch per unit effort over time. The authors are solely responsible for the content and functionality of these materials. Queries (other than absence of the material) should be directed to the corresponding author.
(DOCX)
