# Supplementary material for: Characterising Wildlife Trade Market Supply-Demand Dynamics
Source: PLoS One. 2016 Sep 15;11(9):e0162972. doi: 10.1371/journal.pone.0162972 (PMC5024990; doi:10.1371/journal.pone.0162972)
Supplement: S1 Appendix — (DOCX) [file pone.0162972.s001.docx]

S1 Appendix: Hunter survey instrument

| **B** | **Hunting** |  |  |  |  |
| --- | --- | --- | --- | --- | --- |
|  | Individual Number: | Date: |  |  |  |
|  | Location: | Time: |  |  |  |
| **DEMOGRAPHICS** |  |  |  |  |  |
| **1** | Sex |  |  |  |  |
| **2** | Age |  |  |  |  |
| **3** | Highest Educational level |  |  |  |  |
| **4** | Ethnicity |  |  |  |  |
| **5** | How many year in village |  |  |  |  |
| **6** | What are the livelihood activities carried out? |  |  |  |  |
| **7** | Do you own any farm land? | Yes | No |  |  |
| **8** | Rank livelihood activities as income source |  |  |  |  |
| **9** | How long have you been a hunter | 1. less than 5 | 2. less then 10 | 3. More |  |
| **10** | How comfortable do you consider yourself? | struggling | coping | comfortable | well-off |
| **HUNTING** | |  |  |  |  |
| **11** | Do you hunt all year round? | Yes | No |  |  |
| a | If not what months do you hunt |  |  |  |  |
|  | why? |  |  |  |  |
| b | What months do you not hunt |  |  |  |  |
|  | why? |  |  |  |  |
| **12** | When do you hunt | Day | Night |  |  |
| **13** | How many hours do you hunt on average? |  |  |  |  |
| **14** | What equipment / hunting strategy do you use? | Guns | Trap (forest) | Trap (field) | dogs |
| **15** | Rank most commonly used strategy | Guns | Trap (forest) | Trap (field) | dogs |
| **16** | Number of owned hunting equipments | Guns | Trap (forest) | Trap (field) | dogs |
| **17** | What do you spoend most money on? | bullets | Guns | Traps | transport |
| **18** | What are the reasons for pursuing hunting? (rank) | Income | Food | Crop Pests | Other |
| **19** | What are the peak months for hunting? |  |  |  |  |
|  | why? |  |  |  |  |
| **20** | What are the low months for hunting? |  |  |  |  |
|  | why? |  |  |  |  |
|  | **Heavy Hunting period** |  |  |  |  |
| **21** | Number of hunting days/week for this period |  |  |  |  |
| **22** | Number of caught animals/ week for this period |  |  |  |  |
| **23** | Where do you hunt during this period | Farm | Fallow land | Secondary forest | Primary Forest |
| **24** | Which species do you catch most? |  |  |  |  |
| **25** | Which species do you earn most money from? |  |  |  |  |
| **26** | How far do you travel to hunt in this this period? |  |  |  |  |
| **27** | Mode of Transport | Walking | Car | Bike | other |
| **28** | Rank own game that is: |  |  |  |  |
| a | Sold locally |  |  |  |  |
| b | Sold to Kumasi / Large town market |  |  |  |  |
| d | Consumed by the household |  |  |  |  |
|  | **Low hunting period** |  |  |  |  |
| **29** | Number of hunting days/week for this period |  |  |  |  |
| **30** | Number of caught animals/ week for this period |  |  |  |  |
| **31** | Where do you hunt during this period | Farm | Fallow land | Secondary forest | Primary Forest |
| **32** | Which species do you catch most? |  |  |  |  |
| **33** | Which species do you earn most money from? |  |  |  |  |
| **34** | How far do you travel to hunt in this this period? |  |  |  |  |
| **35** | Mode of Transport | Walking | Car | Bike | other |
| **36** | Rank own game that is: |  |  |  |  |
| a | Sold locally |  |  |  |  |
| b | Sold to Kumasi / Large town market |  |  |  |  |
| c | Consumed by the household |  |  |  |  |
| **TRADITION AND GENERAL** | |  |  |  |  |
| **37** | Do you use hunting to support your family during times of hardship? | |  |  |  |
| **38** | Any species that are no longer hunted? |  |  |  |  |
| **39** | Do you percieve rising fuel prices as a disincentive to hunting? (maybe ask inrelation to transport and city markets? | | | | |
| **40** | During the rainy season is it more difficult to hunt? |  |  |  |  |
|  | Are fires a problem in the dry season? |  |  |  |  |
| **SPECIFICS** | |  |  |  |  |
| **41** | When did you last go hunting? |  |  |  |  |
| a | What did you catch |  |  |  |  |
|  | How far did you travel |  |  |  |  |
|  | Where did you go |  |  |  |  |
| **Hunter consumption/trade** | |  |  |  |  |
| **42** | Do you ever buy bushmeat, or only eat what you catch? |  |  |  |  |
| **43** | What species is your favourite to eat? |  |  |  |  |
| **44** | What species do you eat most often? |  |  |  |  |
| **45** | Would you want your son to be a hunter? |  |  |  |  |
|  | why? |  |  |  |  |
