# Supplementary material for: Characterising Wildlife Trade Market Supply-Demand Dynamics
Source: PLoS One. 2016 Sep 15;11(9):e0162972. doi: 10.1371/journal.pone.0162972 (PMC5024990; doi:10.1371/journal.pone.0162972)
Supplement: S2 Appendix — (DOCX) [file pone.0162972.s002.docx]

S2 Appendix: Urban consumer survey instrument.

| **B** | **Consumer** |  |  |  |
| --- | --- | --- | --- | --- |
|  | Individual Number: | Date: |  |  |
|  | Location: | Time: |  |  |
| **DEMOGRAPHICS** |  |  |  |  |
| **1** | Sex |  |  |  |
| **2** | Age |  |  |  |
| **3** | Tribe |  |  |  |
| **4** | What is your main source of income |  |  |  |
| **THE QUESTIONS** | |  |  |  |
| **5** | What type of meat (inc fish) do you eat most of? (Rank) | Bushmeat (which) | Livestock (which) | Fish |
| **6** | Of all meat (inc fish) what is your favourite? (Rank) | Bushmeat (which) | Livestock (which) | Fish |
| **7** | How often do you eat fish? | Daily | Weekly | Monthly |
| **8** | How often do you eat bushmeat? | Daily | Weekly | Monthly |
| **9** | Where do you buy your bushmeat | Market | Chopbar | Other |
| **10** | If fish was cheap at the market, I would buy more fish and less bushmeat |  |  |  |
| **11** | If the price of bushmeat increased, I would still buy bushmeat. |  |  |  |
| **12** | Do you feel that bushmeat is culturally important? |  |  |  |
