# Supplementary material for: Characterising Wildlife Trade Market Supply-Demand Dynamics
Source: PLoS One. 2016 Sep 15;11(9):e0162972. doi: 10.1371/journal.pone.0162972 (PMC5024990; doi:10.1371/journal.pone.0162972)
Supplement: S3 Appendix — (DOCX) [file pone.0162972.s003.docx]

S3 Appendix: Trader survey instrument

| **D** | **CHOP BAR OWNER** |  |  |  |  |  |
| --- | --- | --- | --- | --- | --- | --- |
|  | Individual Number: | Date: |  |  |  |  |
|  | Location: | Time: |  |  |  |  |
| **DEMOGRAPHICS** |  |  |  |  |  |  |
| **1** | Sex |  |  |  |  |  |
| **2** | Age |  |  |  |  |  |
| **3** | Highest Educational level |  |  |  |  |  |
| **4** | Ethnicity |  |  |  |  |  |
| **5** | How many year in village |  |  |  |  |  |
| **6** | What are the livelihood activities carried out? | Farming | Fishing |  |  |  |
| **7** | Do you own any farmland? | Yes | No |  |  |  |
| **8** | Rank livelihood activities by income |  |  |  |  |  |
| **9** | How long have you been a trader | 1. less than 5 | less then 10 | 3. More |  |  |
| **10** | How comfortable do you consider yourself? | struggling | coping | comfortable | well-off |  |
| **CHOPPING** | |  |  |  |  |  |
|  | **General** |  |  |  |  |  |
| **10** | What is your daily income from trading? |  |  |  |  |  |
| **11** | Do you sell other types of meat |  |  |  |  |  |
| **12** | What do you sell most of (all meat) |  |  |  |  |  |
| **13** | What do you make most of your money from (all meat) |  |  |  |  |  |
| **14** | Where do you source your meat? (non-bushmeat) |  |  |  |  |  |
|  | **Bushmeat** |  |  |  |  |  |
| **15** | Do you sell bushmeat all year round | Yes | No |  |  |  |
| a | if no what months do you trade bushmeat |  |  |  |  |  |
| **16** | What is the most valuable species |  |  |  |  |  |
| **17** | What is the cheapest species |  |  |  |  |  |
| **18** | What are the peak months for trading bushmeat |  |  |  |  |  |
|  | why? |  |  |  |  |  |
| **19** | What are the low months? |  |  |  |  |  |
|  | why? |  |  |  |  |  |
|  | **Peak Season** |  |  |  |  |  |
| **20** | How often do you sell bushmeat? | daily | weekly | monthly | rarely | Never |
| **21** | What do you sell most of in this period? (Rank) |  |  |  |  |  |
| **22** | What do you make most money from during this period? |  |  |  |  |  |
| **23** | Where you buy your bushmeat from (Rank) |  |  |  |  |  |
| a | a) Farmers b) Hunters c) Traders d) Other |  |  |  |  |  |
| **24** | Who do you sell your meat to (Rank) |  |  |  |  |  |
| a | Large Town Markets (which ones) |  |  |  |  |  |
| b | Local Market |  |  |  |  |  |
| c | Households |  |  |  |  |  |
| d | Own consumption |  |  |  |  |  |
| **25** | What species are harvested most frequently? |  |  |  |  |  |
| **26** | What species provides the most income? |  |  |  |  |  |
|  | **Low Trading months** |  |  |  |  |  |
| **27** | How often do you sell bushmeat? | daily | weekly | monthly | rarely | Never |
| **28** | What do you sell most of in this period? (Rank) |  |  |  |  |  |
| **29** | What do you make most money from during this period? |  |  |  |  |  |
| **30** | Where you buy your bushmeat from (Rank) |  |  |  |  |  |
| a | F a) Farmers b) Hunters c) Traders d) Other |  |  |  |  |  |
| **31** | Who do you sell your meat to (Rank) |  |  |  |  |  |
| a | Large Town Markets (which ones) |  |  |  |  |  |
| b | Local Market |  |  |  |  |  |
| c | Households |  |  |  |  |  |
| d | Own consumption |  |  |  |  |  |
| **32** | What species are harvested most frequently? |  |  |  |  |  |
| **33** | What species provides the most income? |  |  |  |  |  |
| **SPECIFICS** | |  |  |  |  |  |
| **34** | In the last week how many animals were traded? |  |  |  |  |  |
| b | From whom goods predominantly bought |  |  |  |  |  |
| c | To who predominantly sold |  |  |  |  |  |
| **35** | What species were traded? |  |  |  |  |  |
| **PAST CHNAGES** | |  |  |  |  |  |
| **36** | Has the amount of bushmeat sold in the village changed | Increased | Decreased | Stayed same |  |  |
| **37** | Has the composition of species changed? | Y | N |  |  |  |
| **38** | What species are no longer present? | Table 6 |  |  |  |  |
| **39** | Has the price of bushmeat changed? | Increased | Decreased | Stayed same |  |  |
|  | Why? |  |  |  |  |  |
