# Supplementary material for: Characterising Wildlife Trade Market Supply-Demand Dynamics
Source: PLoS One. 2016 Sep 15;11(9):e0162972. doi: 10.1371/journal.pone.0162972 (PMC5024990; doi:10.1371/journal.pone.0162972)
Supplement: S5 Appendix — (DOCX) [file pone.0162972.s005.docx]

S5 Appendix: Comparison of changes in market composition (measured in terms of numbers of carcasses entering the market per day) in 1990and 2011 at the Atwemonom Market. Data are presented for the 9 species which were most commonly traded in 1990. Falconer (1992) surveyed 12 days in April, 9 days in May and 6 in June. Out study (2011) surveyed 6 days in May. **Codes:** D – decrease, I – Increase in relative abundance between surveys.

| Species | 1990 | | | 2011 | | |
| --- | --- | --- | --- | --- | --- | --- |
|  | % | Rank | Chg. | % | Rank | Chg. |
| Grasscutter | 48.3 | 1 | - | 62.4 | 1 | - |
| Maxwell duiker | 14.2 | 2 | - | 5.0 | 5 | D |
| Bushbuck | 10.6 | 3 | - | 6.0 | 4 | D |
| Black duiker | 6.9 | 4 | - | 0.7 | 8 | D |
| Royal antelope | 5.4 | 5 | - | 2.6 | 7 | D |
| Red flanked duiker | 4.0 | 6 | - | 0.2 | 12 | D |
| Giant rat | 3.8 | 7 | - | 9.6 | 2 | I |
| Brush-tailed porcupine | 3.7 | 8 | - | 4.3 | 6 | D |
| Ground squirrel | 0.4 | 9 | - | 7.2 | 3 | I |
| **Proportion of total trade** | **97%** |  |  | **98%** |  |  |
