# Supplementary material for: Characterising Wildlife Trade Market Supply-Demand Dynamics
Source: PLoS One. 2016 Sep 15;11(9):e0162972. doi: 10.1371/journal.pone.0162972 (PMC5024990; doi:10.1371/journal.pone.0162972)
Supplement: S6 Appendix — (DOCX) [file pone.0162972.s006.docx]

S6 Appendix: Summary of the one-week survey of the Atwemonom bushmeat market in June 2011

| **Species** | **Latin Name** | **Total Number** | **Total Weight** | **Price per kilo 2011 price** |
| --- | --- | --- | --- | --- |
| Grasscutter | *Thryonomys swinderianus* | 260 | 1226.4 | 8.71 |
| Giant Rat | *Cricetomys* gambinus | 40 | 58.7 | 5.70 |
| Ground Squirrel | *Xerus* spp. | 30 | 54.3 | 3.84 |
| Bushbuck | *Tragelaphus scriptus* | 25 | 1139 | 4.13 |
| Maxwell Duiker | *Cephalophus maxwelli* | 21 | 175.5 | 6.26 |
| B.T Porcupine | *Atherurus africanus* | 18 | 80.3 | 8.09 |
| Royal Antelope | *Neotragus pygmaeus* | 11 | 35.2 | 6.67 |
| Black Duiker | *Cephalophus niger* | 3 | 77 | 4.67 |
| Dwarf Mongoose | *Helogale parvula* | 1 | 3.5 | 2.29 |
| Long Nosed Mongoose | Herpestes naso | 5 | 2.2 | 1.18 |
| Bay Duiker | *Cephalophus dorsalis* | 1 | 14 | 7.14 |
| Red Flanked Duiker | *Cephalophus rufilatus* | 1 | 9 | 5.56 |
| Mona Monkey | *Cercopithecus mona* | 1 | 2.5 | 6.00 |
