# Supplementary material for: Characterising Wildlife Trade Market Supply-Demand Dynamics
Source: PLoS One. 2016 Sep 15;11(9):e0162972. doi: 10.1371/journal.pone.0162972 (PMC5024990; doi:10.1371/journal.pone.0162972)

S7 Appendix: Proportion of hunter reports citing particular species as being present or absent in their catch. “Present” refers to species caught frequently; “Absent” refers to species that used to be caught frequently but are now rare or absent entirely. Species ordered by decreasing body size. Data on body size calculated from (McNamara 2015)

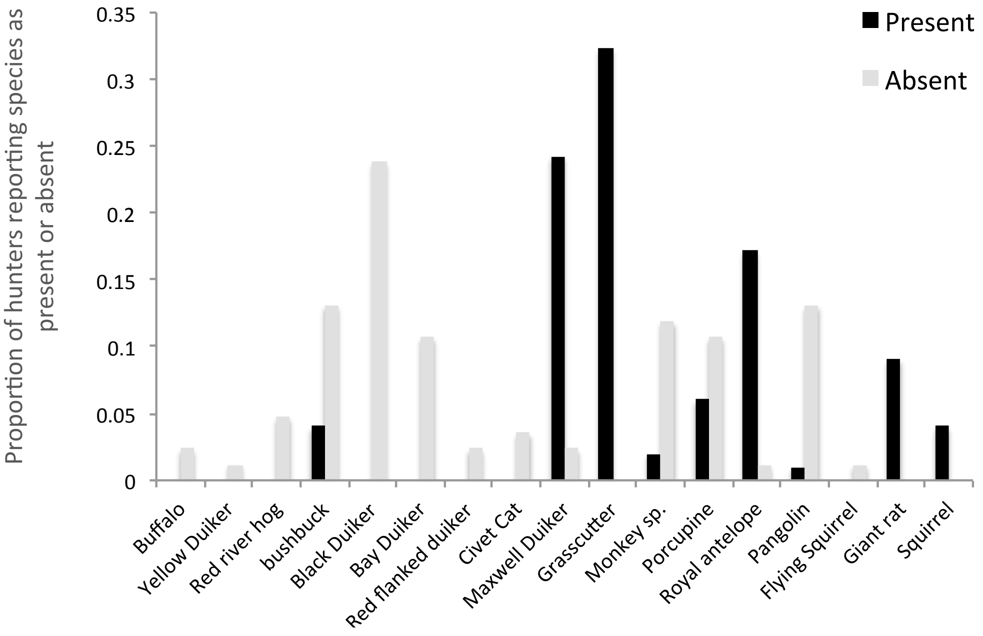

Supplement: S7 Appendix — (PDF) [file pone.0162972.s007.pdf]
