# Supplementary material for: Characterising Wildlife Trade Market Supply-Demand Dynamics
Source: PLoS One. 2016 Sep 15;11(9):e0162972. doi: 10.1371/journal.pone.0162972 (PMC5024990; doi:10.1371/journal.pone.0162972)
Supplement: S8 Appendix — The authors are solely responsible for the content and functionality of these materials. Queries (other than absence of the material) should be directed to the corresponding author. (DOCX) [file pone.0162972.s008.docx]

S8 Appendix: Summary of average hunting trip length and catch per unit effort over time. All study locations were in the Kumasi area. Figures given in parentheses are standard deviations. * Decline in catch per unit effort is significant to the 95% level.

| Period | Average hunting trip length (hrs)  *(S.D)* | Catch per trip (number of animals per trip)  *(S.D)* | Catch per unit effort (number of animals per hour)  *(S.D)* |
| --- | --- | --- | --- |
| 1982  *(Dei 1989)* | 3.6 | NA | NA |
| 1993  *(Ntiamoa-Baidu 1998)* | 4.4 | NA | NA |
| 2002  (Crookes *et al.* 2007) | 5.6 *(3.3)* | 1.97 *(1.2)* | 0.35 *(0.15)* |
| 2011  (Alexander *et al.* 2014) | 7.7 *(3.1)* | 1.95 *(1.5)* | 0.19 *(0.12)** |
